# Simple and User-Friendly Methodology for Crystal Water Determination by Quantitative Proton NMR Spectroscopy in Deuterium Oxide

**DOI:** 10.1021/acs.analchem.3c03689

**Published:** 2023-11-03

**Authors:** Tuulia Tynkkynen, Maria Vassaki, Tommi E. Tiihonen, Vesa-Pekka Lehto, Konstantinos D. Demadis, Petri A. Turhanen

**Affiliations:** † School of Pharmacy, Biocenter Kuopio, University of Eastern Finland, Yliopistonranta 8, FI-70211 Kuopio, Finland; ‡ Crystal Engineering, Growth and Design Laboratory, Department of Chemistry, University of Crete, Heraklion GR-71003, Crete, Greece; § Department of Technical Physics, University of Eastern Finland, Yliopistonranta 8, FI-70211 Kuopio, Finland

## Abstract

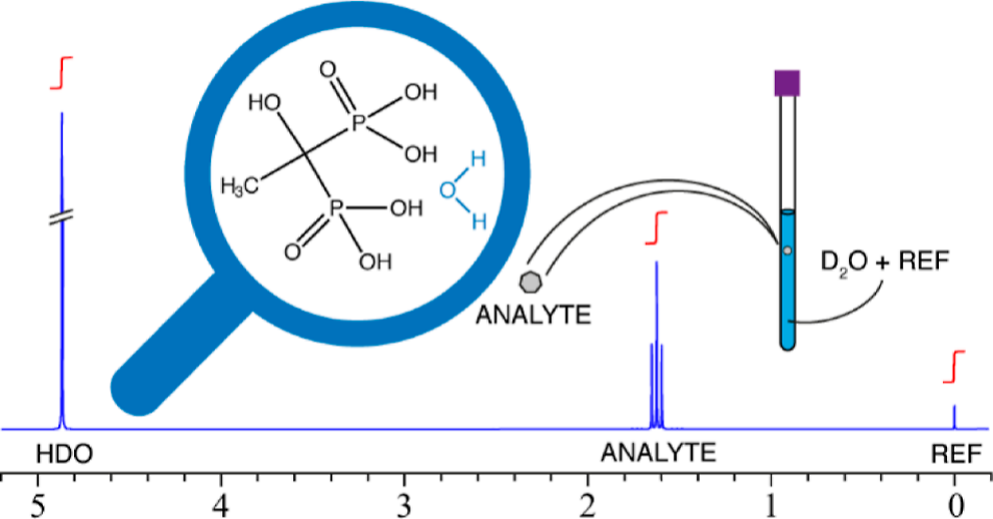

In drug research and development, knowledge of the precise structure of an active ingredient is crucial. However, it is equally important to know the water content of the drug molecule, particularly the number of crystal waters present in its structure. Such knowledge ensures the avoidance of drug dosage and formulation errors since the number of water molecules affects the physicochemical and pharmaceutical properties of the molecule. Several methods have been used for crystal water measurements of organic compounds, of which thermogravimetry and crystallography may be the most common ones. To the best of our knowledge, solution-state NMR spectroscopy has not been used for crystal water determination in deuterium oxide. Quantitative NMR (qNMR) method will be presented in the paper with a comparison of single-crystal X-ray diffraction and thermogravimetric analysis results. The qNMR method for water content measurement is straightforward, reproducible, and accurate, including measurement of ^1^H NMR spectrum before and after the addition of the analyte compound, and the result can be calculated after integration of the reference compound, analyte, and HDO signals using the given equation. In practical terms, there is no need for weighing the samples under study, which makes it simple and is a clear advantage to the current determination methods. In addition, the crystal structures of two model bisphosphonates used herein are reported: that of monopotassium etidronate dihydrate and monosodium zoledronate trihydrate.

The term “crystal water” refers to water molecules present in a compound’s crystal structure. Water is commonly incorporated into the structure upon crystallization from aqueous solutions, and compounds incorporating crystal water molecules are called hydrates. It is often important to know the crystal water content of the compound under study, especially in the case of potential drug candidates entering in vitro and in vivo studies. Interactions with water can affect the functionality of the compounds, and the hydrate forms commonly have different physicochemical and pharmaceutical properties than the corresponding anhydrous forms.^1^ Water in the crystal lattice increases plasticity of the crystals and enhances tabletability.^2,3^ Additionally, the crystal water content affects the total molecular weight of the compound and is needed for the calculation of the drug dosage. For example, one of the early generation bisphosphonate (BP) drugs, (1-hydroxyethan-1,1-diyl)bis(phosphonic acid) a.k.a. etidronic acid, is crystallized as a monohydrate. The molecular weights of the monohydrate and anhydrous forms are 224.05 and 206.03 g/mol, respectively. If the drug dose is calculated ignoring the water molecule, then there will be a ca. 8% error in the drug dosage. The error increases when a larger number of crystal water molecules are present in the solid form of the compound, highlighting the importance of precisely knowing the number of crystal water molecules in the drug’s molecular unit.

A plethora of methods have been used for water content measurements of organic compounds including thermogravimetric analysis (TGA), dynamic vapor sorption (DVS), Karl Fischer (KF) titration or Karl Fischer oven (KF-oven), single-crystal X-ray diffraction (SCXRD), powder X-ray diffraction (PXRD), solid-state NMR spectroscopy (ssNMR), Fourier-transform infrared (FT-IR), and Raman spectroscopy.^4,5^ TGA and DVS give information about the mass change (loss) of the sample upon controlled heating, and the water content of the analyte can be subsequently calculated.^4^ Thermogravimetry can often distinguish between free and bound water.^6^ However, in some metal-phosphonate systems, crystal and metal-bound water molecules are removed in overlapping steps; hence, the separation is difficult. Thermogravimetry can also be used in combination with mass spectrometry^6^ or with differential scanning calorimetry (DSC)^5^ to identify the volatile matter during the heating process. The KF titration method is based on the reaction of iodine and sulfur dioxide in the presence of water using methanol or diethylene glycol monoethyl ether as solvents.^5^ The KF titration is not suitable for substances that release their water very slowly or only at high temperatures, are insoluble in the solvents used in the method, or contain functional groups (especially aldehydes and ketones) that can react with the reagents used in the KF test.^5^ Some of these problems can be overcome by using the KF-oven method, in which the water is first removed in an oven and the released moisture is directed into a titration cell, where the water determination is performed using KF titration.^5^ However, the oven can be heated only up to 250 °C, causing underestimated findings in the case of water of crystallization for some substances. By using SCXRD, it is possible to determine accurate atom positions and the method gives information about both the number and location of water molecules within the crystal structure.^7^ ssNMR methodologies utilize samples crystallized under deuterium oxide vapor and are often combined with quantum chemistry calculations.^8^ The combination of ssNMR and computational methods is called “NMR crystallography”, and the method is primarily used to solve molecular structures in the solid state, but it is also capable of differentiating hydrate and anhydrous forms of a crystal structure.^4,9^ FT-IR and Raman spectroscopies are complementary methods, and they can identify hydrates from the signal caused by the hydroxyl group of H_2_O.^4,10^ Most of these methods necessitate accurate weighing, are time-consuming, require special expertise of the method/equipment in question, and/or destroy the sample material.

Liquid-state NMR spectroscopy is a versatile method suitable for various types of applications, e.g., structure determination, in situ monitoring, quantification of impurities, or quantitative analysis of mixtures.^11−14^ NMR spectroscopy is also increasingly being applied in pharmaceutical research and industry.^15,16^ Importantly, most modern drug synthesis laboratories have NMR equipment for structure and purity analysis, and the same instrumentation can also be used for other applications. Proton (^1^H) NMR spectroscopy is well-suited for water determination due to its quantitative nature: ^1^H NMR signal areas are directly proportional to the number of nuclei giving rise to the signal.^12,17^ Important criteria for the proper application of quantitative NMR (qNMR) spectroscopy include the high solubility of the analyte in the solvent in question, and the presence of some detectable nucleus, which is often hydrogen.^18^ Additionally, several acquisition parameters need to be optimized to obtain quantitative results.^11,17,19^ For example, the repetition time (also called recycling time) between pulses needs to be ≥ 5 × *T*_1_ of the slowest relaxing nucleus, and the number of scans must be suitable to reach adequate signal-to-noise ratio for reliable quantification.^12,20^ A 90-degree pulse is the best choice for qNMR but also shorter pulses can be used if the available measurement time is limited.^19^

In the present study, we introduce a novel, simple, and user-friendly method for crystal water determination of water-soluble compounds by liquid-state ^1^H NMR spectroscopy. The method is applied to several commercial or synthesized “model” compounds, and the NMR results are compared to those obtained with TGA and/or SCXRD. Several of the tested compounds are BPs that are well-known drugs used for bone-related diseases, such as osteoporosis.^21^ BPs are often soluble only in water, and they can contain a variable number of crystal waters in their structure.

## Materials and Methods

### Chemicals and Reference Solutions

Commercial compounds used in the water content measurements: adenosine-5′-triphosphate (ATP) disodium salt hydrate (Grade ≥99%, from microbial, Sigma), sodium acetate trihydrate (pro analysis, Merck), sodium acetate anhydrous (analytical reagent, Riedel-de Haen), magnesium acetate (pro analysis, Merck), citric acid monohydrate (analytical reagent grade, ≥99.8%, Fisher Chemical), trisodium citrate dihydrate (analytical reagent, Riedel-de Haen), trisodium citrate 5.5-hydrate (extra pure, Merck), gallic acid monohydrate (ACS reagent, ≥98.0%, Sigma-Aldrich). Etidronic acid monohydrate was synthesized and characterized as described elsewhere.^22^ Rubidium etidronate, Rb[CH_3_C(OH)(PO_3_H_2_)(PO_3_H)]·2H_2_O, was crystallized according to the literature.^23^ Monosodium zoledronate trihydrate, {Na_2_[(C_4_H_6_N_2_)C(OH)(PO_3_H)_2_]_2_(H_2_O)_3_}·3H_2_O, was crystallized by adjusting the pH of an aqueous solution of zoledronic acid to the value of ∼5 with NaOH, followed by layering with methanol. Monopotassium etidronate dihydrate, {K[(CH_3_)C(OH)(PO_3_H_2_)(PO_3_H)]}·2H_2_O, was crystallized by adjusting the pH of an aqueous solution of etidronic acid to the value of ∼5 with KOH, followed by layering with methanol. TSP (3-trimethylsilylpropionic-2,2,3,3-*d*_4_ acid sodium salt) and deuterium oxide (99.8%) were ordered from Eurisotop. Formic acid (AnalaR Normapur, 99.7%) was obtained from Fisher Chemical. For the NMR measurements, reference solutions containing either 1.5 mM TSP or 20 mM formic acid in deuterium oxide were prepared.

### NMR Sample Preparation

NMR samples were prepared by pipetting ca. 500 μL of a reference solution into a 5 mm NMR tube. After the ^1^H NMR measurement of the reference solution, a small amount of the compound under study was placed in the same NMR tube using an appropriate spatula. The solution was gently mixed by inverting the NMR tube several times until all solids were dissolved, and the ^1^H NMR measurement was repeated using the same parameters as for the reference sample.

The repeatability of the method was tested by preparing some NMR samples in duplicates, and the relative standard deviation was calculated for the duplicate results.

### ^1^H NMR Spectroscopy

The samples were measured on a Bruker AVANCE III HD (Bruker BioSpin GmbH, Karlsruhe, Germany) 600 MHz NMR spectrometer equipped with a cryogenically cooled TCI cryoprobe using a zg pulse sequence containing a 90° excitation pulse. The acquisition parameters included 8 scans (NS), 84132 data points (TD), spectral width (SW) 20.0246 ppm, receiver gain (RG) 2, acquisition time (AQ) 3.5 s, and relaxation delay (D1) 60 s. Some of the spectra were measured also with AQ 8 s and/or D1 200 s. The spectra were measured at a 295 K temperature.

The free induction decays were multiplied with an exponential line-broadening function (LB = 0.5 Hz) before Fourier transformation. Phase correction and integration of signals were performed manually by the Topspin 3.6.2 version (Bruker BioSpin GmbH, Karlsruhe, Germany). The integral of the reference compound (either TSP or formic acid) was always set to 1.0. Water content of the samples was calculated using eq 1.
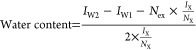
1where *I*_W2_ = integral of the HDO signal in the spectrum containing the compound X. *I*_W1_ = integral of the HDO signal in the spectrum containing only the reference solution. *N*_ex_ = number of exchangeable protons in the structure of the compound X. *I*_X_ = integral of the compound X signal. *N*_X_ = number of hydrogens yielding the ^1^H NMR signal of the compound X.

Estimates of *T*_1_ times of formic acid and HDO were determined by measurements using the t1ir1d pulse program. The first spectrum was measured using a very short delay (d7 = 0.000003 s), and the phases were manually corrected to get all the NMR peaks negative. The measurement parameters included NS 1, TD 81920, SW 21.036 ppm, RG 57, AQ 3.24 s, and D1 150 s. Several other spectra were measured with otherwise the same parameters except that the d7 delay was gradually increased until the peak of interest was nulled. Below the correct d7 the signal is negative, whereas above the correct d7, the signal is positive. The *T*_1_ time for the signal of interest is d7 for the null divided by the natural logarithm of 2.

### Thermogravimetric Analysis

The samples were measured with a NETZSCH TG 209 F1 Libra thermogravimetric analyzer in an open Al_2_O_3_ pan to determine the content of the crystal water in the compounds. The samples were first heated isothermally at 70 °C for 30–120 min to remove the “free” surface water and further heated with a heating rate of 10 °C/min up to 300 °C. The mass loss between the end of the isothermal step and 200 °C was considered as crystal water removed from the compound. All measurements were carried out under a 20 mL/min N_2_ gas flow with three replicates.

### Single-Crystal X-ray Diffraction

Measured crystals were prepared under inert conditions and immersed in perfluoropolyether as a protecting oil for manipulation. Suitable crystals were mounted on MiTeGen Micromounts, and these samples were used for data collection. Data for the compounds monopotassium etidronate dihydrate and monosodium zoledronate trihydrate were collected with a Bruker D8 Venture diffractometer with graphite monochromated Cu Kα (λ = 1.54178 Å). The data were processed with the APEX3 suite [Bruker APEX3. APEX3 V2019.1; Bruker-AXS: Madison, WI, USA, 2019.]. The structures were solved by intrinsic phasing using the ShelXT program,^24^ which revealed the position of all non-hydrogen atoms. These atoms were refined on *F*^2^ by a full-matrix least-squares procedure, using the anisotropic displacement parameter.^25^ All hydrogen atoms were located in difference Fourier maps and included as fixed contributions riding on attached atoms with isotropic thermal displacement parameters 1.2 or 1.5 times those of the respective atom. The Olex2 software was used as a graphical interface.^26^ Molecular graphics were generated using Mercury.^27^ The crystallographic data for the reported structures were deposited with the Cambridge Crystallographic Data Center. CCDC numbers: for monopotassium etidronate dihydrate 2277643 and for monosodium zoledronate trihydrate 2277644. The data can be obtained free of charge at https://www.ccdc.cam.ac.uk/structures.

## Results and Discussion

### Choice of a Reference Compound and Workflow for the qNMR Method

The ^1^H NMR spectrum shows separate signals from all of the chemically inequivalent hydrogens present in a sample at concentrations above the detection limit. Deuterium oxide (D_2_O) was chosen as the solvent because compounds containing crystal water commonly contain polar (e.g., –OH and/or –NH_*x*_) or ionic (e.g., phosphonate) groups and are water-soluble. It is important to note that deuterium oxide always contains a small amount of HDO and will give a residual signal at about 4.7 ppm. This residual HDO content of the solvent must be considered in the crystal water calculations. Our proposed approach is to perform the measurements in a deuterium oxide solution containing a reference compound. The reference compound can be arbitrarily selected but should give a signal that does not overlap with the HDO or the analyte signals to enable accurate integration. We tested the performance of two reference compounds, TSP and formic acid. TSP is commonly used both as a chemical shift reference and a concentration reference and is commonly available in NMR laboratories. TSP gives only one singlet signal at 0 ppm that does not typically overlap with the other signals. Formic acid is a simple and commonly available compound that gives one singlet signal at approximately 8.46 ppm. The use of formic acid is recommended particularly if the analyte compound must be recovered after the water content measurement because formic acid can be easily evaporated, in contrast to TSP, which will remain with the sample.

**Scheme 1:**
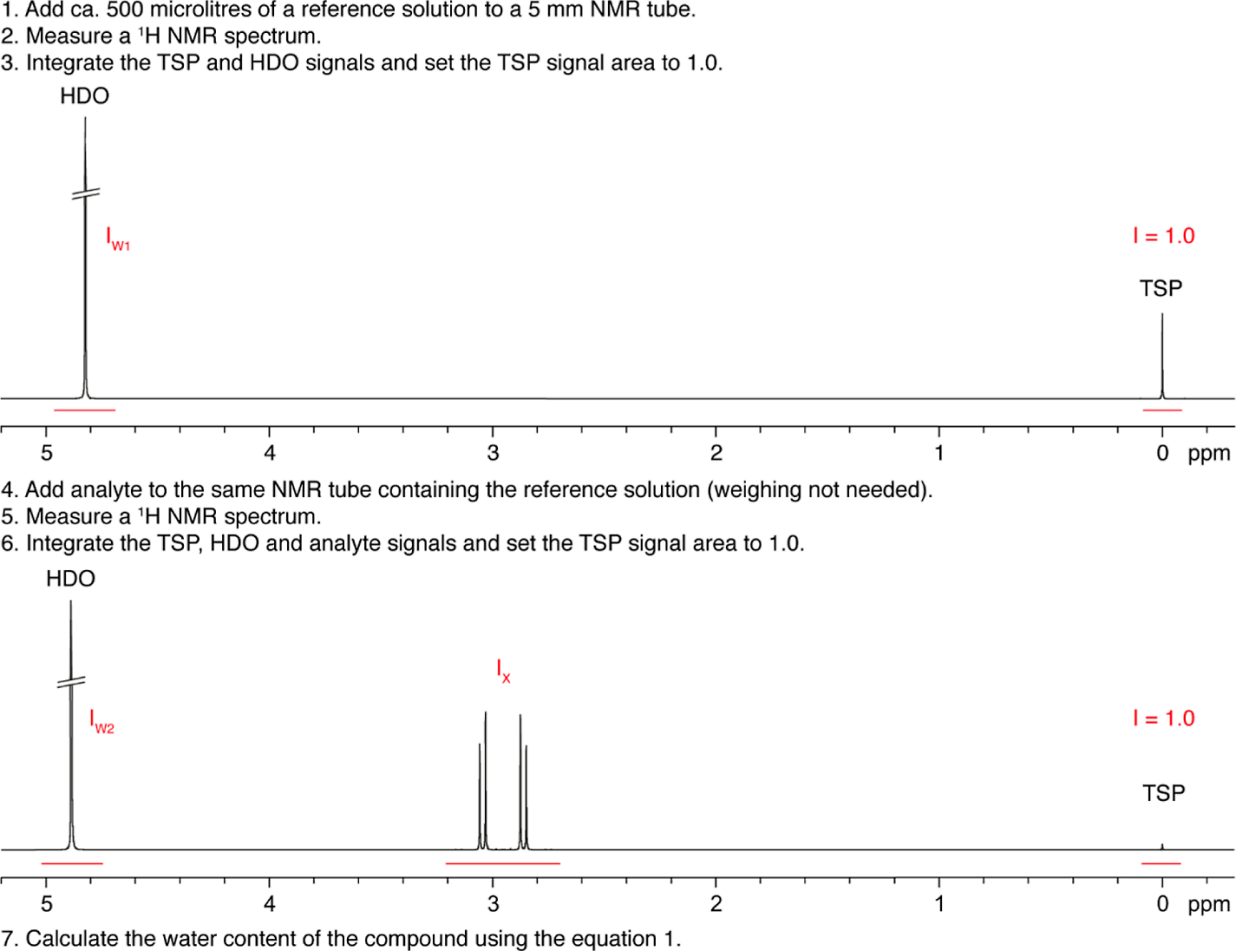
Workflow of Crystal Water Determination In this example case, TSP and citric acid were used as a reference and an analyte compound, respectively. Both citrate signals at 2.85 and 3.05 ppm were integrated and included in the calculation because their ^13^C satellite signals were partially overlapped.

The workflow of the protocol for crystal water determination is shown in Scheme 1. The method is designed to be simple and easy to perform and does not require the expertise of an NMR specialist or even weighing the compound under analysis. The protocol requires ^1^H NMR spectra to be recorded before and after the addition of the analyte compound, and the result can be calculated after integration of the reference compound, analyte, and HDO signals using eq 1. As an example, the ^1^H NMR spectra of a reference solution and a reference solution after addition of etidronic acid monohydrate together with the crystal water calculation are shown in Figure S1. Commonly, it is sufficient to integrate only one of the analyte signals. In certain cases, some of the analyte signals overlap with the HDO signal. This can be taken into account in the calculation by subtracting the integral value corresponding to the number of protons overlapping the water signal, as demonstrated for monosodium zoledronate trihydrate in Figure S2. Alternatively, it would be possible to use deconvolution for signal area determination instead of integration.^28,29^

### Robustness of ^1^H NMR Measurement Parameters

Small molecules have long longitudinal relaxation times (*T*_1_). The relaxation times of HDO and formic acid protons were determined to be ∼9 and 11 s, respectively. For quantitative measurements, the repetition time (acquisition time + relaxation delay) between excitation pulses is advised to be ≥5 times the longest *T*_1_ to allow ≥99.3% relaxation.^12^ Thus, the repetition time must be ≥45 s to allow HDO protons to be adequately relaxed. It is also important to use a long-enough acquisition time to collect the fully decayed free induction decay (FID).^30^ However, acquisition time that is too long collects only noise. Commonly, the acquisition time is set to 5–8 s for quantitative measurements.^30^

The robustness of the measurement parameters was tested by changing the acquisition time and the relaxation delay resulting in repetition times of 63.5, 68, 203.5, and 208 s. Crystal water determinations for citric acid monohydrate and sodium acetate trihydrate with two different acquisition times and relaxation delays, as well as using either TSP or formic acid as reference compounds are presented in Table 1. The difference between results obtained with acquisition times of 3.5 and 8 s is negligible, and thus, the acquisition time of 3.5 s is already sufficient. According to our results (Table 1), the relaxation delay of 60 s is adequate also when using formic acid as the reference compound even though the *T*_1_ time is a bit longer for formic acid than for HDO. This is because the reference compound signal does not need to be fully relaxed, as the same measurement parameters are used in both measurements, and the reference compound signal is always set to a constant value. The most important thing is to get the HDO and analyte compound protons relaxed because their integrals are used for the water content calculation. The measurement time for one spectrum using an acquisition time of 3.5 s and a relaxation delay of 60 s is ca. 13 min.

**Table 1:** Crystal Water Determination Data for Commercially Available Compounds Obtained Using Variable Measurement Parameters and Reference Compounds

analyte compound	reference compound	acquisition time (s)	FID resolution (Hz)	relaxation delay (s)	determined crystal waters N(H_2_O)
citric acid·1H_2_O	TSP	3.5	0.286	60	1.02
				200	1.02
		8.0	0.125	60	1.02
				200	1.02
citric acid·1H_2_O	formic acid	3.5	0.286	60	1.03
				200	1.04
		8.0	0.125	60	1.04
				200	1.04
sodium acetate·3H_2_O	TSP	3.5	0.286	60	3.01
				200	3.02
		8.0	0.125	60	3.01
				200	3.01
sodium acetate·3H_2_O	formic acid	3.5	0.286	60	3.03
				200	3.05
		8.0	0.125	60	3.04
				200	3.05

### Required Analyte Concentration

NMR samples from several compounds including a variable number of exchangeable protons were prepared with varying concentrations in order to evaluate any concentration effects. Variable-concentration experiments were performed for commercial compounds (Table 2), and the samples were prepared without exact weighing by adding low, average, and high amounts of analyte compounds to the NMR tubes to keep the method as simple as possible during all stages of the protocol. Surprisingly, all low-concentration samples gave results that were systematically ∼10% higher (per crystal water molecule) than the actual crystal water in the compounds. For the higher-concentration samples, the results were more accurate (Table 2). Concentrations in mmol/L and mg/mL units were calculated from the ^1^H NMR spectra, and according to the results obtained, a reasonable concentration for accurate water content measurements is around 100 mmol/L or ca. 10–20 mg/mL (5–10 mg in 0.5 mL reference solution in a 5 mm NMR tube); however, this somewhat depends on the individual compound. The number of exchangeable protons does not seem to influence the method’s accuracy. Notably, ATP (and analogues) can exist in various hydrate forms, and it is pivotal to check the crystal water content prior to preparing ATP solutions with certain concentrations.

**Table 2:** Crystal Water Determination Data for Commercially Available Compounds Using Various Analyte Concentrations

analyte compound	N(Ex)^a^	determined crystal waters N(H_2_O)	analyte concentration *c* (mmol/L)	analyte concentration *c* (mg/mL)
citric acid·1H_2_O	4	1.06	129.73	27.26
		1.05	263.42	55.36
		1.02	469.75	98.72
trisodium citrate·2H_2_O	1	2.36	13.22	3.89
		2.09	43.04	12.66
		2.02	173.79	51.11
sodium acetate·3H_2_O	0	3.29	24.28	3.30
		3.05	105.98	14.42
		3.02	430.11	58.53
sodium acetate anhydrous	0	0.14	64.74	5.31
		0.05	260.93	21.41
		0.06	746.05	61.20
gallic acid·1H_2_O	4	1.35	27.52	5.18
		1.08	36.79	6.92
		0.95	39.77	7.48
magnesium acetate tetrahydrate^b^	0	2.05	101.45	21.76
		2.04	212.89	45.65
		2.02	313.59	67.25
disodium ATP·2H_2_O	6	2.08	29.31	17.21

a Number of exchangeable protons in the structure.

b Two crystal water molecules per acetate molecule.

### Comparison with Other Methods and Reproducibility

In order to compare our qNMR methodology with those of two commonly used water determination methods, a set of compounds were also measured by TGA and SCXRD (Table 3). The compounds included two commercially available citrates and four BPs. The TGA data for all six compounds are shown in Figure S3. According to the TGA and qNMR results, both citrates contain two crystal waters. Trisodium citrate 5.5-hydrate had been stored unopened for at least 15 years before the qNMR and TGA measurements, and thus, the crystal water content of the compound stabilized to two per citrate molecule.

**Table 3:** Comparative Crystal Water Contents of Selected Compounds Determined by qNMR, TGA, and SCXRD

compound	N(Ex)^a^	NMR			TGA		SCXRD
		determined crystal waters N(H_2_O)	RSD %^b^	analyte concentration *c* (mmol/L)^c^	analyte concentration N(H_2_O)	RSD %^b^	determined crystal waters
trisodium citrate·2H_2_O	1	2.02	n.d.	174	1.98	0.05	2^33^
trisodium citrate·5.5H_2_O	1	2.02	1.07	150/184	1.98	0.30	5.5^34^
etidronic acid·H_2_O	5	1.06	0.19	97/137	1.01	4.45	1
monopotassium etidronate·2H_2_O	4	1.98	1.60	87/115	1.85	1.68	2
monorubidium etidronate·2H_2_O	4	2.08	3.24	21/95	2.01	0.45	2
monosodium zoledronate·3H_2_O	4	2.30	n.d.	40	1.96	0.77	3

a Number of exchangeable protons in the structure.

b Relative standard deviation (RSD %) was calculated for the replicates using the equation RSD % = 100 × SD/μ, where SD is the sample standard deviation and μ is the sample mean.

c Concentrations of duplicates reported separately. n.d. = not determined.

**Figure 1:**
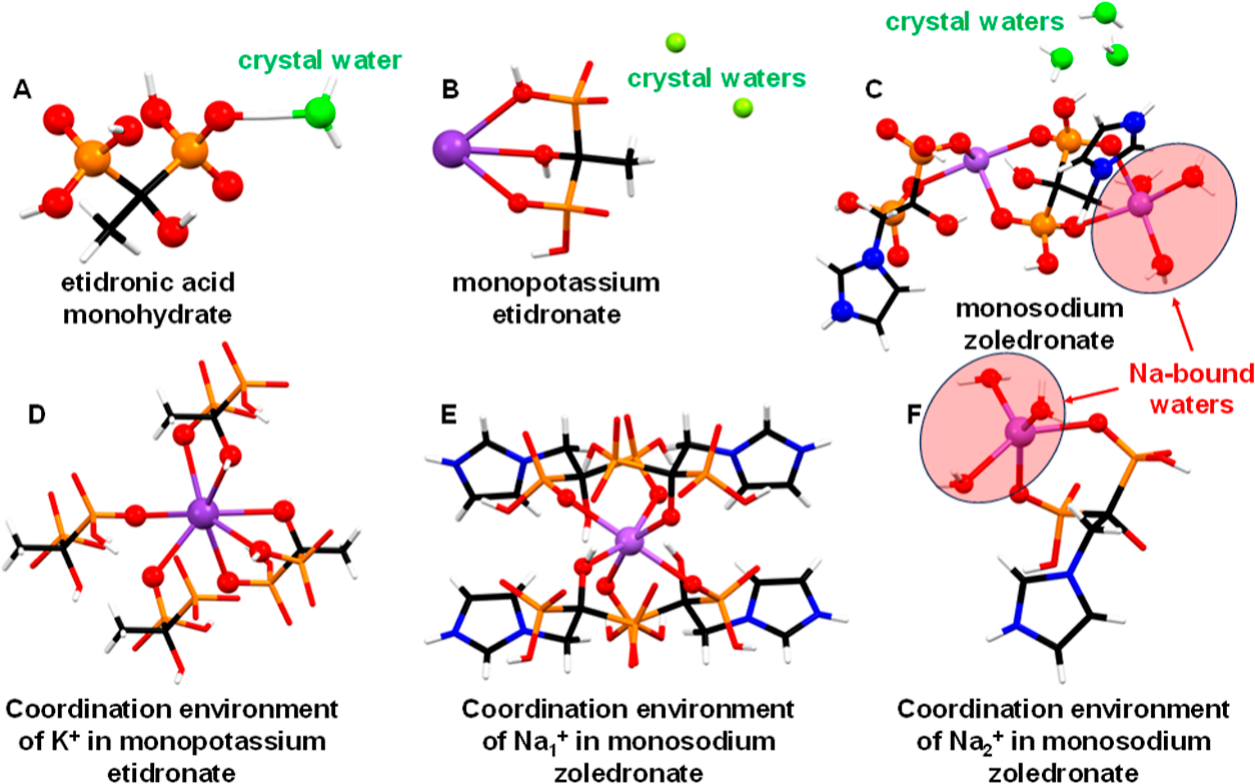
(A) Asymmetric unit of etidronic acid monohydrate. (B) Asymmetric unit of monopotassium etidronate. (C) Asymmetric unit of monosodium zoledronate. (D) Coordination environment of the K^+^ center in the structure of monopotassium etidronate. (E,F) Coordination environment of the two different Na^+^ centers in the structure of monosodium zoledronate. Color codes: metal centers (K^+^ or Na^+^), magenta; O, red; P, orange; C, black; H white. Crystal waters are shown in green.

Structural elucidation for all four BPs included in this work was performed by SCXRD. Notably, the structures of the new compounds monopotassium etidronate and monosodium zoledronate are reported here for the first time. The structure of etidronic acid monohydrate has been published before.^31^ Its asymmetric unit is shown in Figure 1A. There is one crystal water.

The etidronate anion in the structure of monopotassium etidronate is found in its monodeprotonated state, and it is charge-balanced by the K^+^ cation (Figure 1B). The K^+^ center is 7-coordinated in a capped trigonal prismatic geometry (Figure 1D), surrounded by three types of etidronate anions. The first forms a tris-chelating arrangement (via the C–OH group and two protonated P–OH moieties, one from each phosphonate). The second type of etidronate binding is terminal, with two P–OH groups from two different etidronate ligands binding to the K^+^ center. Lastly, one etidronate molecule binds to the K^+^ center in a bidentate fashion (via the C–OH group and a protonated P–OH group). The K–O bond distances are in the range of 2.749–3.080 Å, within the expected range observed in other K-phosphonate compounds.^7,32^ There are two crystal waters in the structure (Figure 1B), which are hydrogen-bonded to neighboring phosphonate oxygens.

The structure of monosodium zoledronate (Figure 1C) exhibits additional complexity, as there are two crystallographically distinct Na^+^ centers (Figure 1E,F). The first (Na1) is found in a distorted octahedral coordination environment bound by four phosphonate oxygens and two –OH groups (in a trans arrangement). The second (Na2) has a peculiar distorted trigonal pyramidal geometry, and it is bound by two phosphonate oxygens in a bidentate fashion, as well as by three water molecules in a meridional arrangement. There are three crystal waters in its structure. They form multiple hydrogen bonds with phosphonate oxygens and other water molecules.

In the case of etidronic acid, the TGA showed that the crystal water is loosely bound because it was removed already during the 70 °C isothermal phase, which made it difficult to observe but was not a problem with the qNMR method. The results for monopotassium etidronate dihydrate and monorubidium etidronate dihydrate were well in line between all three methods, giving two crystal waters for both compounds. Regarding monosodium zoledronate trihydrate, the SCXRD analysis was performed a couple of years prior to the present qNMR and TGA measurements and showed three crystal waters per zoledronate molecule. However, according to the TGA and qNMR results, the compound currently contains only two crystal waters per zoledronate molecule. Similarly, as with trisodium citrate 5.5-hydrate, all the crystal waters in monosodium zoledronate trihydrate are not stable. Both trisodium citrate 5.5-hydrate and monosodium zoledronate trihydrate have lost some of the crystal water during long storage and/or handling. Hence, for commercially available compounds, even though the water content of the compound is usually described on the container label, the only way to be confident about the water content of the analyte is to measure the water content before its use, and the qNMR method described here allows for fast and simple determination.

It is worth mentioning that the BP samples were sent from Heraklion (Crete, Greece) to Kuopio (Finland) during the month of February. The first ^1^H NMR measurements of monosodium zoledronate trihydrate were performed a few days after the sample’s arrival. The measurements showed the presence of three crystal waters (specifically, 2.99 and 3.10 in two different measurements with varying concentrations). The next qNMR measurement was performed at least three months later together with TGA. Both methods showed the presence of two crystal waters. The air moisture levels in Kuopio (Finland) and in Heraklion (Crete, Greece) at the end of February are different, being much lower in Finland than in Crete where the monosodium zoledronate trihydrate was crystallized and measured by SCXRD. It can be speculated that the storage of the monosodium zoledronate trihydrate at ambient temperature in lower moisture conditions may have induced the loss of one water molecule.

The majority of the qNMR water content determinations for the compounds shown in Table 3 were performed in duplicates, and the relative standard deviations were within 0.19–3.24%. The corresponding relative standard deviations for the TGA data were 0.05–4.45%, indicating that the repeatabilities of the NMR and TGA methods are on the same level. Higher RSD % for the monorubidium etidronate is caused by low concentrations of the samples since the lower analyte concentrations tend to overestimate the results as discussed above. When the concentrations of the replicates are at least ca. 100 mmol/L, the relative standard deviations are well below 2%.

### Strengths and Limitations

The qNMR methodology described in this paper is novel, fast, nondestructive, and accurate. The sample preparation is simple because (a) no weighing of the sample is required (adding ∼5–15 mg of the analyte compound using an appropriate spatula to the NMR tube), and (b) the volume of the reference solution does not have to be precise (just pipetting around 0.5 mL of the reference solution using Pasteur pipet to the NMR tube). If the concentration of the analyte appears to be too low, it is possible to simply add more solids to the same NMR tube and repeat the measurement. Furthermore, NMR spectroscopy enables the identification of possible organic impurities present in the sample with the same measurement. Additionally, the availability of basic NMR equipment in modern drug synthesis laboratories enables an easy adaptation of the qNMR methodology for water determinations.

Interestingly, recent technical developments on benchtop NMR spectrometers have made NMR spectrometers more accessible since the cost of such instrumentation is considerably lower than that of a high-field spectrometer.^35^ Benchtop NMR spectrometers have lower sensitivity and resolution than their higher-field counterparts, but they can be utilized for various purposes for which high sensitivity and resolution are not required. Detection and quantification limits for benchtop NMR spectroscopy are at a millimolar range that would be adequate for crystal water determination because the preferred analyte concentration is ca. 100 mM. In the future, the applicability of benchtop NMR for crystal water determination will be explored in practice.

It must be acknowledged that the qNMR method cannot distinguish between crystal water and surface water. If the analyte compound is prone to adsorb surface water, then it is advised to dry the analyte compound under high vacuum to remove surface water prior to the qNMR analysis. This handling should not remove the crystal water if the sample is not heated, unless the crystal water is exceptionally loosely bound. This was tested to the etidronic acid monohydrate, and crystal water remained in the structure. Also, the analyte compound must have at least one nonexchangeable proton and produce a signal that does not overlap with the HDO or the reference compound signals.

## Conclusions

Accounting for the simplicity, repeatability, accuracy, nondestructiveness, and speed, the qNMR method described herein appears to be a convenient and user-friendly alternative to currently existing methods for crystal water determination. It is anticipated that this method will be broadly adopted in the fields of structural and medicinal/pharmaceutical chemistry, where water content measurements are crucial.

## Supporting Information Available

Examples of ^1^H NMR spectra of a reference solution, etidronic acid monohydrate, and monosodium zoledronate with crystal water calculation and thermogravimetric data used to determine the crystal water content of the compounds (PDF)
Crystallographic data for the compound monopotassium etidronate(CIF)
Crystallographic data for the compound monosodium zoledronate (CIF)

## Supplementary Material

ac3c03689_si_001.pdf

ac3c03689_si_002.cif

ac3c03689_si_003.cif

## References

[ref1] Okeyo P. O.; Ilchenko O.; Slipets R.; Larsen P. E.; Boisen A.; Rades T.; Rantanen J. Imaging of dehydration in particulate matter using Raman line-focus microscopy. Sci. Rep. 2019, 9, 7525 10.1038/s41598-019-43959-0. 31101829 PMC6525166

[ref2] Sun C. C.; Grant D. J. W. Improved tableting properties of p-hydroxybenzoic acid by water of crystallization: a molecular insight. Pharm. Res. 2004, 21, 382–386. 10.1023/B:PHAM.0000016272.81390.b4. 15032322

[ref3] Reddy C. M. Plasticity enhancement in pharmaceutical drugs by water of crystallization: unusual slip planes. IUCrJ 2019, 6, 505–506. 10.1107/S205225251900890X. PMC6608643 31316794

[ref4] Jurczak E.; Mazurek A. H.; Szeleszczuk Ł.; Pisklak D. M.; Zielińska-Pisklak M. Pharmaceutical hydrates analysis - Overview of methods and recent advances. Pharmaceutics 2020, 12, 959 10.3390/pharmaceutics12100959. 33050621 PMC7601571

[ref5] Hinz D. C. Evaluation of methods for the determination of water in substances with unknown chemical and thermal behaviour. J. Pharm. Biomed. Anal. 2007, 43, 779–783. 10.1016/j.jpba.2006.08.002. 16956740

[ref6] Komatsu H.; Yoshii K.; Okada S. Application of thermogravimetry to water-content determinations of drugs. Chem. Pharm. Bull. 1994, 42, 1631–1635. 10.1248/cpb.42.1631.

[ref7] Salcedo I. R.; Colodrero R. M. P.; Bazaga-García M.; Vasileiou A.; Papadaki M.; Olivera-Pastor P.; Infantes-Molina A.; Losilla E. R.; Mezei G.; Cabeza A.; Demadis K. D. From light to heavy alkali metal tetraphosphonates (M = Li, Na, K, Rb, Cs): cation size-induced structural diversity and water-facilitated proton conductivity. CrystEngComm 2018, 20, 7648–7658. 10.1039/C8CE01351A.

[ref8] Gowda V.; Laitinen R. S.; Telkki V.-V.; Larsson A.-C.; Antzutkin O. N.; Lantto P. DFT calculations in the assignment of solid-state NMR and crystal structure elucidation of a lanthanum(III) complex with dithiocarbamate and phenanthroline. Dalton Trans. 2016, 45, 19473–19484. 10.1039/C6DT03705D. 27891541

[ref9] Bryce D. L. NMR crystallography: structure and properties of materials from solid-state nuclear magnetic resonance observables. IUCrJ 2017, 4, 350–359. 10.1107/S2052252517006042. PMC5571798 28875022

[ref10] Van Eerdenbrugh B.; Taylor L. S. Application of mid-IR spectroscopy for the characterization of pharmaceutical systems. Int. J. Pharm. 2011, 417, 3–16. 10.1016/j.ijpharm.2010.12.011. 21167267

[ref11] Giraudeau P. Quantitative NMR spectroscopy of complex mixtures. Chem. Commun. 2023, 59, 6627–6642. 10.1039/D3CC01455J. 37132658

[ref12] Wei R.; Dickson C. L.; Uhrín D.; Lloyd-Jones G. C. Rapid estimation of T_1_ for quantitative NMR. J. Org. Chem. 2021, 86, 9023–9029. 10.1021/acs.joc.1c01007. 34155887

[ref13] Crook A. A.; Powers R. Quantitative NMR-based biomedical metabolomics: Current status and applications. Molecules 2020, 25, 5128 10.3390/molecules25215128. 33158172 PMC7662776

[ref14] Pauli G. F.; Chen S.-N.; Simmler C.; Lankin D. C.; Gödecke T.; Jaki B. U.; Friesen J. B.; McAlpine J. B.; Napolitano J. G. Importance of Purity Evaluation and the Potential of Quantitative ^1^H NMR as a Purity Assay: Miniperspective. J. Med. Chem. 2014, 57, 9220–9231. 10.1021/jm500734a. 25295852 PMC4255677

[ref15] Monakhova Y. B.; Holzgrabe U.; Diehl B. W. K. Current role and future perspectives of multivariate (chemometric) methods in NMR spectroscopic analysis of pharmaceutical products. J. Pharm. Biomed. Anal. 2018, 147, 580–589. 10.1016/j.jpba.2017.05.034. 28583765

[ref16] Sørensen D.; Szabo C.; Nelson M.; Miura T.; Corbett C.; Napolitano J.; Giraudeau P.; Shelbourn T.; Ray J. Quantitative Nuclear Magnetic Resonance (qNMR), a Metrological Method-Proposed Revisions to the USP General Chapters on NMR, Nuclear Magnetic Resonance Spectroscopy 761 and Applications of Nuclear Magnetic Resonance Spectroscopy 1761. https://www.usp.org/sites/default/files/usp/document/workshops/stimuli-article-qnmr.pdf (accessed Aug 8, 2023).

[ref17] Pauli G. F.; Jaki B. U.; Lankin D. C. Quantitative ^1^H NMR: Development and Potential of a Method for Natural Products Analysis. J. Nat. Prod. 2005, 68, 133–149. 10.1021/np0497301. 15679337

[ref18] Jaki B. U.; Bzhelyansky A.; Pauli G. F. Quantitative NMR (qNMR) for pharmaceutical analysis: The pioneering work of George Hanna at the US FDA. Magn. Reson. Chem. 2021, 59, 7–15. 10.1002/mrc.5099. 32910504

[ref19] Bharti S. K.; Roy R. Quantitative 1H NMR spectroscopy. Trends Anal. Chem. 2012, 35, 5–26. 10.1016/j.trac.2012.02.007.

[ref20] Holzgrabe U. Quantitative NMR spectroscopy in pharmaceutical R&D. eMagRes 2015, 4, 45–56. 10.1002/9780470034590.emrstm1399.

[ref21] Malwal S. R.; O’Dowd B.; Feng X.; Turhanen P.; Shin C.; Yao J.; Kim B. K.; Baig N.; Zhou T.; Bansal S.; Khade R. L.; Zhang Y.; Oldfield E. Bisphosphonate-generated ATP-analogs inhibit cell signaling pathways. J. Am. Chem. Soc. 2018, 140, 7568–7578. 10.1021/jacs.8b02363. 29787268 PMC6022752

[ref22] Turhanen P. A.; Vepsäläinen J. J. Strategies for the preparation of (1-Acetyloxyethylidene)-1,1-bisphosphonic acid derivatives. Synthesis 2004, 7, 0992–0994. 10.1055/s-2004-822345.

[ref23] Charpin P.; Lance M.; Nierlich M.; Vigner D.; Lee M.-R.; Silvestre J.-P.; Dao N. Q. Structure du trihydrogéno hydroxy-1-ethanedi(phosphonate)-1,1 de rubidium dihydrate. Acta Crystallogr. 1988, 44, 990–992. 10.1107/s0108270188001283.

[ref24] Sheldrick G. M. SHELXT-integrated space-group and crystal-structure determination. Acta Crystallogr., Sect. A: Found. Adv. 2015, 71, 3–8. 10.1107/S2053273314026370. 25537383 PMC4283466

[ref25] Sheldrick G. M. Crystal structure refinement with SHELXL. Acta Crystallogr., Sect. C: Struct. Chem. 2015, 71, 3–8. 10.1107/S2053229614024218. 25567568 PMC4294323

[ref26] Dolomanov O. V.; Bourhis L. J.; Gildea R. J.; Howard J. A. K.; Puschmann H. OLEX2: A complete structure solution, refinement and analysis program. J. Appl. Crystallogr. 2009, 42, 339–341. 10.1107/S0021889808042726.

[ref27] Macrae C. F.; Bruno I. J.; Chisholm J. A.; Edgington P. R.; McCabe P.; Pidcock E.; Rodriguez-Monge L.; Taylor R.; van de Streek J.; Wood P. A. Mercury CSD 2.0 - New features for the visualization and investigation of crystal structures. J. Appl. Crystallogr. 2008, 41, 466–470. 10.1107/S0021889807067908.

[ref28] Schmid N.; Bruderer S.; Paruzzo F.; Fischetti G.; Toscano G.; Graf D. F. M.; Fey M.; Henrici A.; Ziebart V.; Heitmann B.; Grabner H.; Wegner J.; Sigel R.; et al. Deconvolution of 1D NMR spectra: A deep learning-based approach. J. Magn. Reson. 2023, 347, 107357 10.1016/j.jmr.2022.107357. 36563418

[ref29] Sokolenko S.; Jézéquel T.; Hajjar G.; Farjon J.; Akoka S.; Giraudeau P. Robust 1D NMR lineshape fitting using real and imaginary data in the frequency domain. J. Magn. Reson. 2019, 298, 91–100. 10.1016/j.jmr.2018.11.004. 30530098

[ref30] Schoenberger T. ENFSI working group - guideline for qNMR analysis. https://enfsi.eu/wp-content/uploads/2017/06/qNMR-Guideline_version001.pdf (accessed Aug 8, 2023).

[ref31] Silvestre J.-P.; Dao N. Q.; Heger G.; Cousson A. Refinement by neutron diffraction of the crystal structure of hydroxyethylidene bisphosphonic acid monohydrate: C(CH_3_)(OH)(PO_3_H_2_)_2_·H2O. Phosphorus, Sulfur Silicon Relat. Elem. 2002, 177, 277–288. 10.1080/10426500210223.

[ref32] Bazaga-García M.; Papadaki M.; Colodrero R. M. P.; Olivera-Pastor P.; Losilla E. R.; Nieto-Ortega B.; Aranda M. A. G.; Choquesillo-Lazarte D.; Cabeza A.; Demadis K. D. Tuning proton conductivity in alkali metal phosphonocarboxylates by cation size-induced and water-facilitated proton transfer pathways. Chem. Mater. 2015, 27, 424–435. 10.1021/cm502716e.

[ref33] Fischer A.; Palladino G. Trisodium citrate dihydrate. Acta Crystallogr. 2003, 59, m1080–m1082. 10.1107/S1600536803024395.

[ref34] Voissat B.; Rodier N. Crystal structure of sodium citrate hydrate. Bull. Soc. Chim. Fr. 1986, 4, 522–525.

[ref35] Castaing-Cordier T.; Bouillaud D.; Farion J.; Giraudeau P. Chapter four - Recent advances in benchtop NMR spectroscopy and its applications. Annu. Rep. NMR Spectrosc. 2021, 103, 191–258. 10.1016/bs.arnmr.2021.02.003.

