# Supplementary material for: Simple and User-Friendly Methodology for Crystal Water Determination by Quantitative Proton NMR Spectroscopy in Deuterium Oxide
Source: Anal Chem. 2023 Nov 3;95(46):17020–7. doi: 10.1021/acs.analchem.3c03689 (PMC10666084; doi:10.1021/acs.analchem.3c03689)
Supplement: Supplementary file 1 — ac3c03689_si_001.pdf [file ac3c03689_si_001.pdf]

Supporting Information

A simple and user-friendly methodology for crystal water determination by quantitative proton NMR spectroscopy in deuterium oxide

Tuulia Tynkkynen[1,*], Maria Vassaki[2], Tommi E. Tiihonen[3], Vesa-Pekka Lehto[3], Konstantinos D. Demadis[2] and Petri A. Turhanen[1,*]

[1]*School of Pharmacy, Biocenter Kuopio, University of Eastern Finland, FI-70211, Kuopio, Finland*

[2]*Crystal Engineering, Growth and Design Laboratory, Department of Chemistry, University of Crete, Heraklion Crete, GR-71003, Greece*

[3]*Department of Technical Physics, University of Eastern Finland, FI-70211, Kuopio, Finland*

*Corresponding authors

Tuulia Tynkkynen – tuulia.tynkkynen@uef.fi
Petri A. Turhanen – petri.turhanen@uef.fi

Table of Contents

Figure S1: ^{1}H NMR spectra and crystal water calculation for etidronic acid monohydrate…………………………………………………………….. S3

Figure S2: ^{1}H NMR spectra and crystal water calculation for monosodium zoledronate………………………………………………………………. S4

Figure S3: Thermogravimetric data used to determine the crystal water content of the compounds……………………………………………………………… S5

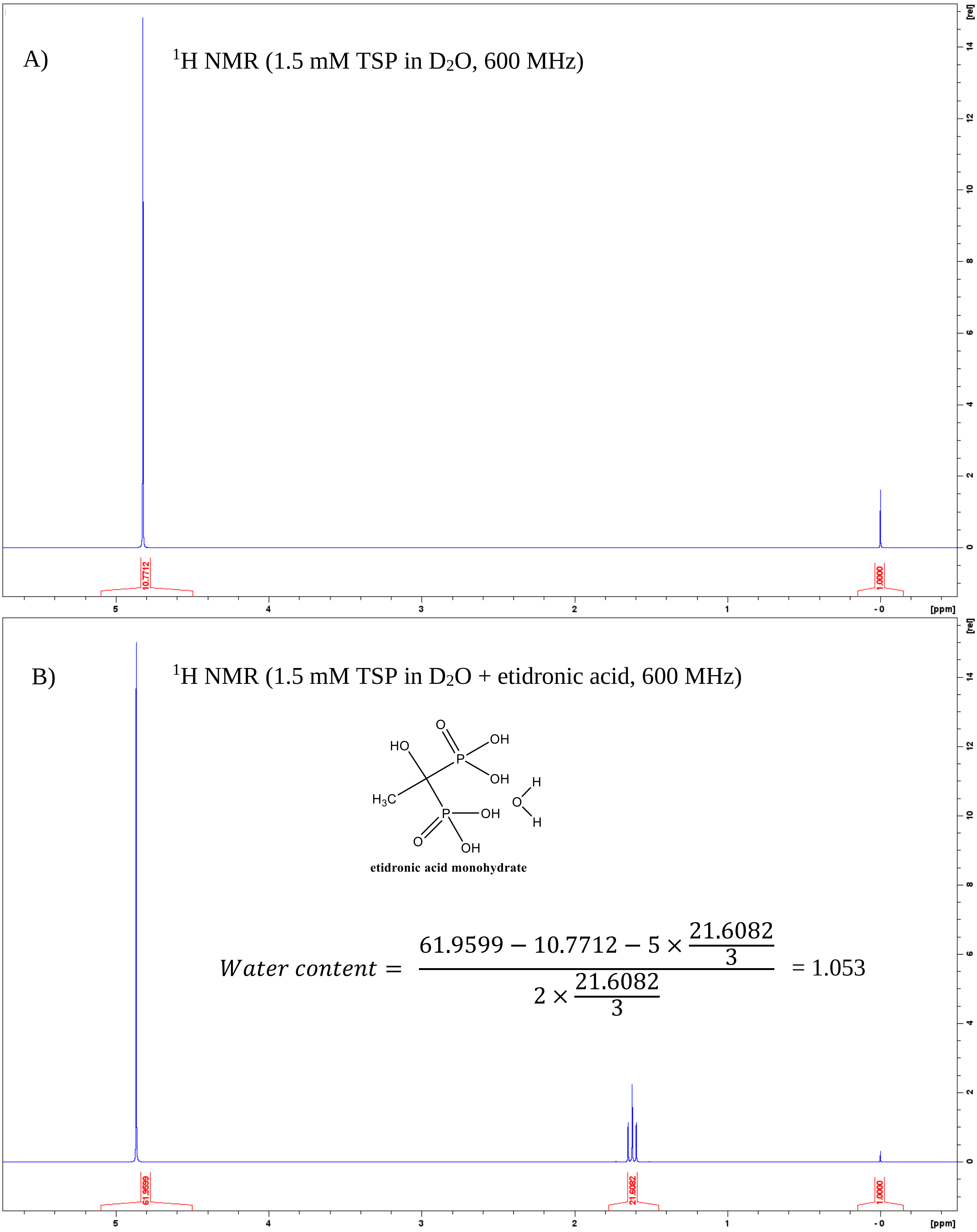

Figure S1. ^{1}H NMR spectra of a reference solution containing TSP (A) and the same solution after addition of etidronic acid monohydrate (B).

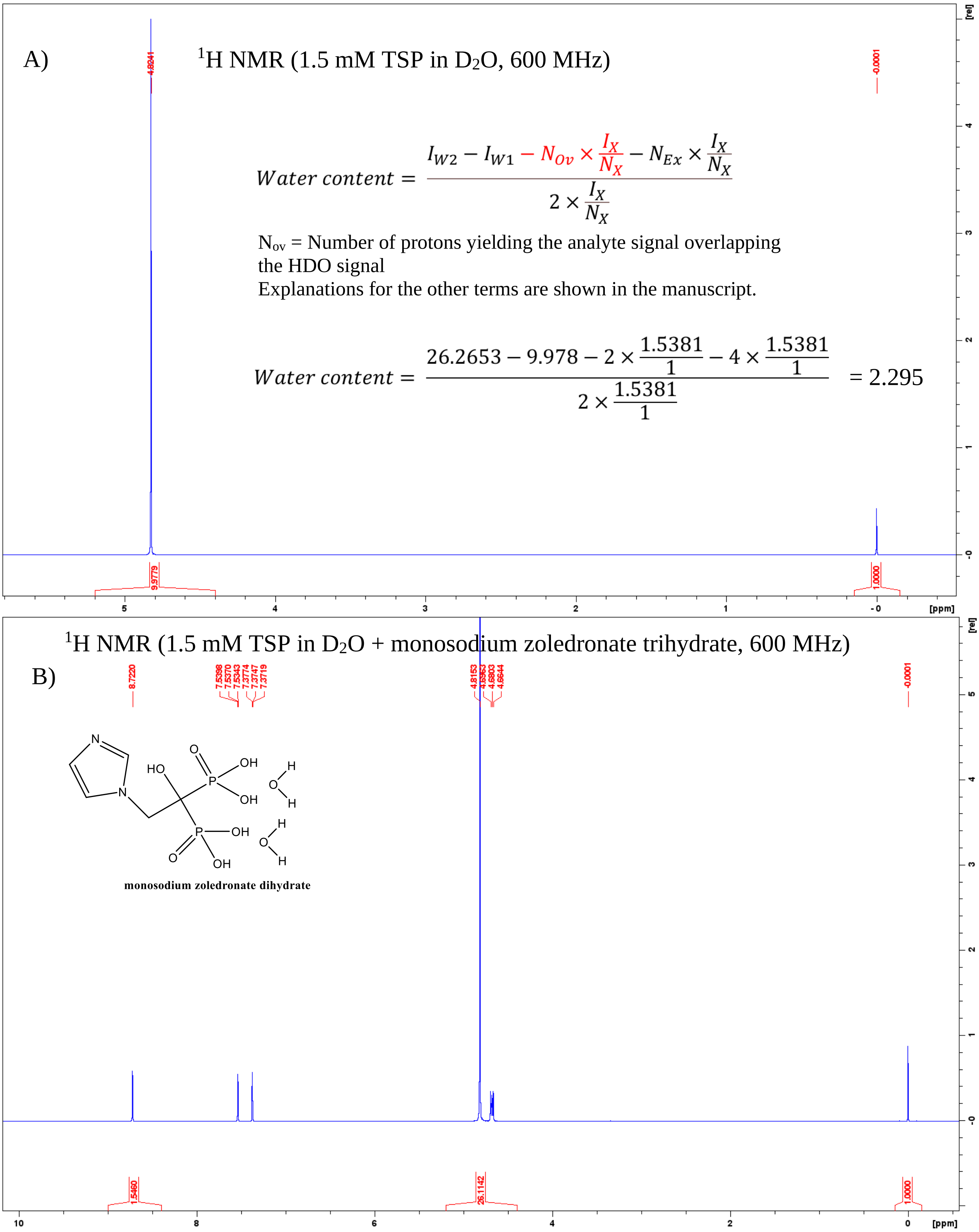

Figure S2. ¹H NMR spectra of a reference solution containing TSP (A) and the same solution after addition of monosodium zoledronate (B). One signal from the analyte is close to the HDO signal and must be integrated together with the HDO signal. The contribution of this analyte signal is subtracted in the calculation. The modified version of the equation 1 (modification illustrated in red) accounting for the overlapping signal is given.

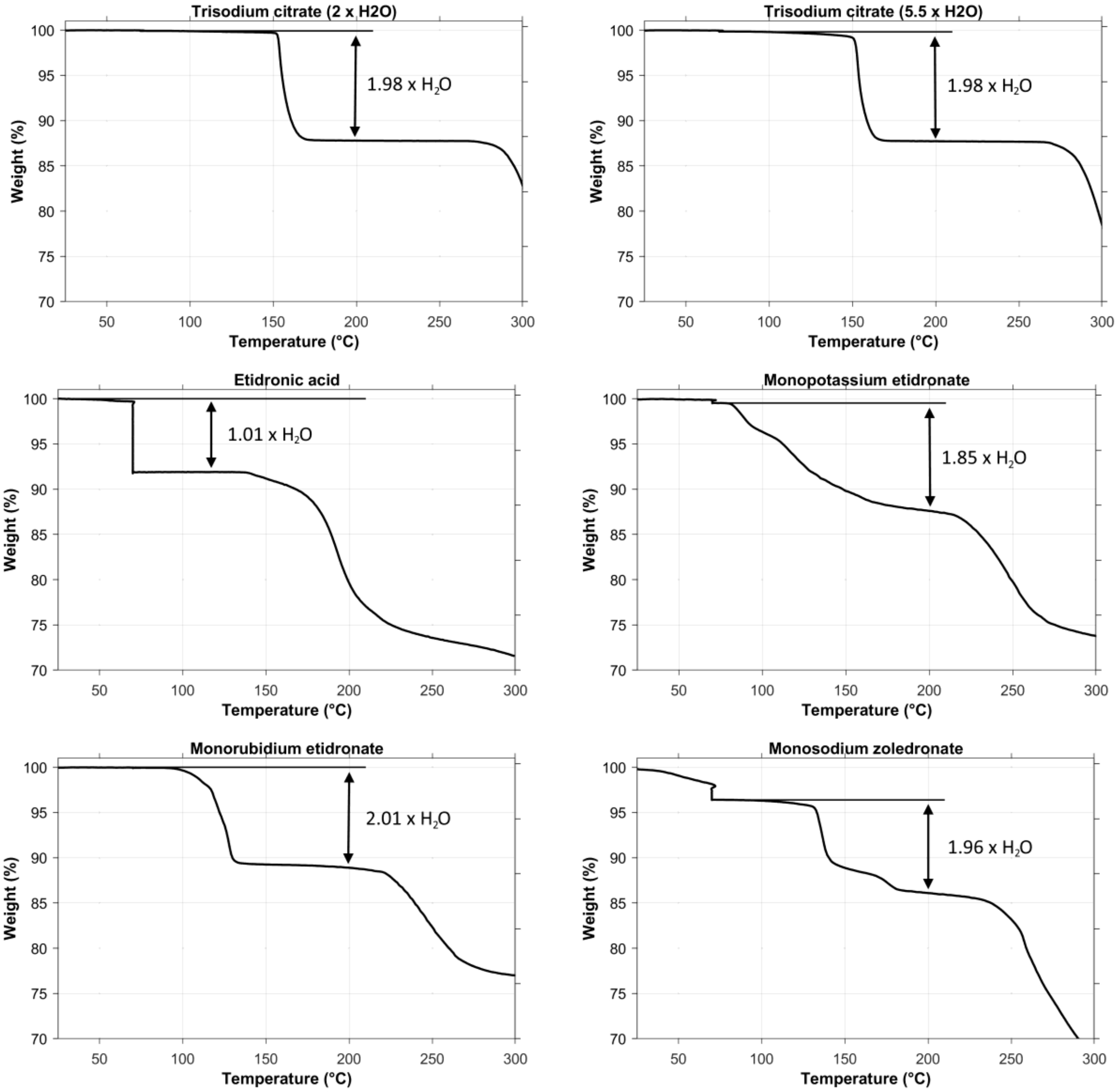

Figure S3. Thermogravimetric data used to determine the crystal water content of the compounds**.**
